# The autoimmune disease risk variant NCF1-His90 is associated with a reduced risk of tuberculosis in women

**DOI:** 10.3389/fimmu.2025.1514296

**Published:** 2025-01-23

**Authors:** Xinjun Hu, Shasha Li, Renliang Huang, Ziwei Fu, Chenyu Ma, Zheng Cheng, Hongjun Hu, Qiaomiao Zhou, Frank Petersen, Xinhua Yu, Junfeng Zheng

**Affiliations:** ^1^ Department of Infectious Diseases, The First Affiliated Hospital of Henan University of Science and Technology, Henan Medical Key Laboratory of Gastrointestinal Microecology and Hepatology, Luoyang, China; ^2^ Institute of Psychiatry and Neuroscience, Xinxiang Medical University, Xinxiang, China; ^3^ Department of Genetics and Prenatal Diagnosis, Hainan Women and Children’s Medical Center, Haikou, Hainan, China; ^4^ Department of Surgical Oncology, Xinxiang Central Hospital, The Fourth Clinical of Xinxiang Medical University, Xinxiang, China; ^5^ Priority Area Chronic Lung Diseases, Research Center Borstel - Leibniz Lung Center, Members of the German Center for Lung Research (DZL), Borstel, Germany

**Keywords:** autoimmune diseases (AD), infectious diseases, neutrophil cytosolic factor 1 (NCF1), genetic association, evolutionary trade-offs, tuberculosis

## Abstract

**Introduction:**

The neutrophil cytosolic factor 1 (*NCF1*) rs201802880 polymorphism is a missense mutation resulting in an amino acid substitution from arginine to histidine at position 90, which impairs the function of NADPH oxidase. This casual variant confers an increased risk for multiple autoimmune disorders, including primary Sjögren’s syndrome and systemic lupus erythematosus. Given the high prevalence of this autoimmune disease risk variant in East Asia, we hypothesized that it may confer an evolutionary advantage by providing protection against infectious diseases.

**Methods:**

To test this hypothesis, we investigated whether the *NCF1* rs201802880 variant offers a protective effect against tuberculosis (TB), a historically significant and deadly infectious disease. Our study included 490 healthy controls and 492 TB patients who were genotyped for the *NCF1* rs201802880 polymorphism.

**Results:**

Our results showed that the *NCF1* rs201802880 AA genotype was associated with a reduced risk of TB in women (OR= 0.25, 95% CI: 0.09-0.68, *p*=0.0023). Additionally, healthy individuals with the NCF1 rs201802880 AA genotype had significantly lower circulating white blood cell (5.56 ± 1.78 vs 6.43 ± 1.59, *p*=0.003) and neutrophil (3.23 ± 1.20 vs 3.74 ± 1.23, *p* = 0.02) counts compared to those with the GG or GA genotypes, with this difference being more pronounced in women than in men.

**Conclusion:**

This study demonstrates that the autoimmune disease-causal NCF1 variant is associated with a protective effect against TB infection.

## Introduction

Genetic association studies have identified the GTF2I-NCF1 intergenic region on chromosome 7 as a significant susceptibility locus for various autoimmune disorders, including primary Sjögren’s syndrome (pSS) (1, 2), systemic lupus erythematosus (SLE) (3, 4), rheumatoid arthritis (5), systemic sclerosis (SSc) (6), and neuromyelitis optica spectrum disorder (NMOSD) (7, 8). In 2017, the causal polymorphism within this susceptibility locus was pinpointed as the neutrophil cytosolic factor 1 (NCF1) rs201802880 G>A variant. This missense mutation results in an amino acid substitution from arginine (Arg) to histidine (His) at position 90 (9, 10). Beyond its association with disease susceptibility, the NCF1 Arg90His variant has been linked to various clinical and immunological features in SLE. These include an earlier age at diagnosis, presence of anti-beta2 glycoprotein I and anticardiolipin antibodies, increased formation of neutrophil extracellular traps (NETs), elevated serum interferon activity, and impaired macrophage efferocytosis (9–12). This association is further supported by experimental data from NCF1-His90 knock-in (KI) mice, which exhibit reduced oxidative burst, diminished macrophage efferocytosis, splenomegaly, increased type I interferon (IFN-I) scores, and higher levels of plasma cells, as well as enhanced Pristane-induced kidney disease compared to wild-type littermates (11).

The NCF1 Arg90 residue is evolutionarily conserved within the p47phox subunit of the phagocyte NADPH oxidase complex. The frequency of the NCF1 rs201802880 A allele varies significantly globally, being less than 0.5% in Caucasian populations, while exceeding 15% in East Asian groups such as Chinese, Japanese, and Korean populations (13). Despite its association with autoimmune disorders, including those linked to infertility and pregnancy loss due to autoantibodies such as anti-beta2 glycoprotein I and anticardiolipin antibodies (14, 15), the variant persists in human populations. This suggests that the NCF1 Arg90His variant may confer an evolutionary advantage, possibly by offering protection against infectious diseases. Indeed, neutrophils with the homozygous AA genotype demonstrate markedly reduced production of reactive oxygen species (ROS) compared to those with GG or GA genotypes (9, 12). Consistent with this, splenocytes from NCF1-His90 KI mice show a reduced capacity for ROS generation (11). Considering the crucial role of ROS in the pathogenesis of tuberculosis (TB) (16), a contagious disease caused by infection with *Mycobacterium tuberculosis* (*Mtb*) bacteria and coexisted with human for more than 40,000 years history (17), we hypothesize that the homozygous AA genotype may confer resistance to TB. This study aims to investigate the relationship between the NCF1 Arg90His variation and susceptibility to tuberculosis.

## Materials and methods

### Patients and control subjects

All patients with tuberculosis (TB) and healthy control subjects were recruited from the First Affiliated Hospital of Henan University of Science and Technology, Luoyang, China. The diagnosis of TB was made in accordance with the Chinese Guidelines for the Diagnosis and Treatment of Tuberculosis (2020 edition) (18). Specifically, individuals meeting any of the following four criteria were classified as having TB: (1) positive sputum smear for acid-fast bacilli; (2) culture of *Mycobacterium tuberculosis* from sputum, bronchoalveolar lavage fluid, or pleural effusion; (3) positive nucleic acid test for *M. tuberculosis* and/or positive culture in sputum, bronchoalveolar lavage fluid, or pleural effusion; (4) positive acid-fast bacilli staining or nucleic acid test for *M. tuberculosis* in lung tissue specimens from the lesion site. Both pulmonary and extrapulmonary TB patients were included in this study. All procedures were conducted in accordance with the principles of the Declaration of Helsinki, and ethical approval for the study protocol was obtained from the Ethics Committee of the First Affiliated Hospital of Henan University of Science and Technology. The ethical approval batch number is 2024-03-K187.

### Data collection

Demographic and clinical data, including sex, age, clinical symptoms, purified protein derivative (PPD) skin test results, erythrocyte sedimentation rate (ESR), treatment regimens, and responses to treatment, were collected from electronic medical records. Hematological parameters, including counts of white blood cells (WBC), neutrophils (NEU), eosinophils (EOS), basophils (BAS), monocytes (MON), lymphocytes (LYM), red blood cells (RBC), and platelets (PLT), were measured for both TB patients and healthy controls using a Sysmex XN-1000 Analyzer (Sysmex, Japan).

### DNA isolation and genotyping

Genomic DNA was extracted from peripheral blood leukocytes using the TaKaRa Blood Genome DNA Extraction Kit (Takara Biotechnology, Dalian Co., Ltd., China) following the manufacturer’s protocol. Genotyping of the *NCF1* rs201802880 G>A polymorphism was conducted using nested PCR followed by a TaqMan assay, as previously described (10). Briefly, a specific *NCF1* fragment was initially amplified through PCR by targeting the GTGT sequence in exon 2 of the gene. The resulting PCR product then served as the template for SNP genotyping using the TaqMan assay.

### Statistical analysis

Statistical analyses were conducted using GraphPad Prism software (version 5.01, GraphPad Software Inc., La Jolla, CA, USA). Hardy–Weinberg equilibrium (HWE) was assessed using Fisher’s exact test, with *p* < 0.05 indicating a deviation from HWE. The Kolmogorov-Smirnov test was used to assess the normality of quantitative variables. For data following a normal distribution, comparisons between two groups were conducted using the Student’s *t*-test. For non-normally distributed data, the Mann-Whitney *U* test was applied. Genotype frequency differences were analyzed using Fisher’s exact test or chi-square test, as appropriate. Five genetic models—co-dominant, dominant, recessive, over-dominant, and additive—were applied for the genetic association analysis using the SNPSTATS program (https://www.snpstats.net/). The optimal inheritance model was determined based on the Akaike Information Criterion (AIC) and Bayesian Information Criterion (BIC), with the model yielding the lowest AIC and BIC values considered the best fit. Statistical significance was defined as *p* < 0.05.

## Results

### Demographic, clinical and laboratory features of patients with active tuberculosis

A total of 492 patients with active TB and 490 healthy control subjects were included in this study. The demographic, clinical, and laboratory characteristics of both groups are summarized in **Table 1**. The average age of TB patients was 45.0 ± 18.6 years, which was approximately 6 years older than that of the control subjects. Compared to the controls, TB patients exhibited higher levels of circulating neutrophils, eosinophils, and monocytes, while levels of lymphocytes and red blood cells were reduced. Among the 492 TB patients, 39 (7.93%) had extrapulmonary tuberculosis. The PPD skin test was administered to 223 patients, with 92.8% testing positive (defined as a reaction >10 mm). All patients received antibiotic treatment, with 54.9% showing a favorable response. Drug resistance was observed in 20.5% of patients, who were resistant to one or more antibiotics. The patient cohort included 196 females and 296 males, while the healthy control group comprised 221 females and 269 males. Stratified analysis revealed that females exhibited lower levels of WBC, neutrophils, eosinophils, basophils, monocytes, and RBC compared to males in both TB patients and healthy individuals. Furthermore, female TB patients demonstrated better treatment responses and a lower incidence of drug resistance compared to male patients (**Table 1**).

**Table 1:** Demographic, clinical and laboratory features of patients with active tuberculosis.

	Healthy controls			TB patients		
	All (n=490)	Male (n=269)	Female^$^ (n=221)	All^§^ (n=492)	Male (n=296)	Female^$^ (n=196)
Age, years (mean ± SD)	38.7 ± 15.8	37.1 ± 15.0	40.6 ± 16.5*	45.0 ± 18.6****	45.8 ± 18.2	43.8 ± 19.0
Hematological parameters						
WBC (10^3^/μL)	6.38 ± 1.64	6.63 ± 1.69	6.06 ± 1.53**	6.33 ± 2.21	6.70 ± 2.19	5.80 ± 2.14****
NEU (10^3^/μL)	3.70 ± 1.23	3.84 ± 1.27	3.53 ± 1.17**	4.10 ± 1.97***	4.39 ± 1.89	3.68 ± 2.01****
EOS (10^3^/μL)	0.14 ± 0.12	0.16 ± 0.13	0.12 ± 0.10***	0.18 ± 0.17****	0.20 ± 0.18	0.15 ± 0.15**
BAS (10^3^/μL)	0.03 ± 0.02	0.04 ± 0.02	0.03 ± 0.02**	0.03 ± 0.02	0.04 ± 0.02	0.03 ± 0.02**
MON (10^3^/μL)	0.36 ± 0.18	0.39 ± 0.19	0.33 ± 0.15**	0.42 ± 0.17****	0.47 ± 0.18	0.35 ± 0.13****
LYM (10^3^/μL)	2.14 ± 0.65	2.18 ± 0.65	2.08 ± 0.64	1.58 ± 0.59****	1.60 ± 0.61	1.58 ± 0.56
RBC (10^6^/μL)	4.78 ± 0.58	5.05 ± 0.55	4.46 ± 0.44****	4.65 ± 0.55***	4.79 ± 0.59	4.45 ± 0.42****
PLT (10^3^/μL)	243.9 ± 63.6	238.5 ± 56.7	250.4 ± 70.6*	244.0 ± 81.3	239.8 ± 83.8	250.0 ± 77.2
Extrapulmonary tuberculosis	–	–	–	39 (7.93%)	18 (6.08%)	21 (10.7%)
PPD above 10 mm	–	–	–	207/223 (92.8%)	118/128 (92.2%)	89/95 (92.7%)
ESR (mm/h)	–	–	–	21.0 (7.0 - 44.0)	21.0 (6.5 - 41.0)	21.0 (8.0 - 48.5)
Treatment with antibiotics	–	–	–	492 (100%)	296 (100%)	196 (100%)
Response to treatment	–	–	–	396 (80.4%)	221 (74.6%)	175 (89.2%)****
Drug resistance	–	–	–	101 (20.5%)	80 (27.0%)	21 (10.7%)****

^§^484 out of 492 patients with follow up data. PPD, purified protein derivative; ESR, erythrocyte sedimentation rate; WBC, white blood cells; NEU, neutrophils; EOS, eosinophils; BAS, basophils; MON, monocytes; LYM, lymphocytes; RBC, Red blood cells; PLT, platelets; ^§^Comparison between TB patients and healthy controls; ^$^Comparison between women and men. Quantitative data following a normal distribution are expressed as mean ± standard deviation (SD), whereas non-normally distributed quantitative data are reported as median (Q1–Q3). Categorical variables are presented as frequency (number of samples) and percentage. *p<0.05, **p<0.01, ***p<0.001 and ****p<0.0001.

### *NCF1* rs201802880 AA genotype confers resistance to tuberculosis in women

To assess the hypothesis that the NCF1 rs201802880 AA genotype offers protection against tuberculosis, we genotyped all 492 TB patients and 490 controls for this polymorphism. The genotype distribution for NCF1 rs201802880 was in Hardy-Weinberg equilibrium for both patient and control groups. Among the five genetic models, the recessive model demonstrated the lowest AIC and BIC values and was therefore selected for the association analysis (**Supplementary Table 1**).

Compared to healthy controls, TB patients exhibited a trend towards a lower frequency of the AA genotype, though this difference was not statistically significant (4.7% vs 6.7%, OR = 0.68, 95% CI: 0.39-1.17, *p* = 0.164) (**Table 2**). Given the association of NCF1 rs201802880 with autoimmune diseases, which predominantly affect women, we further explored the genotype-disease relationship by stratifying the analysis by gender. In women, the frequency of the AA genotype was significantly lower among TB patients compared to controls (2.6% vs 9.5%, OR = 0.25, 95% CI: 0.09-0.68, *p*=0.0023). Conversely, no significant difference was observed in men (**Table 2**).

**Table 2:** Association of NCF1 Arg90His variation with TB.

All subjects	Control (n=490)	TB (n=492)	OR (95% CI)*	*p* value
GG	318 (64.9%)	300 (61.0%)		
GA	139 (28.4%)	169 (34.3%)		
AA	33 (6.7%)	23 (4.7%)	0.68 (0.39-1.16)	0.164
Male	Control (n=269)	TB (n=296)		
GG	181 (67.3%)	182 (61.5%)		
GA	76 (28.2%)	96 (32.4%)		
AA	12 (4.5%)	18 (6.1%)	1.39 (0.66-2.94)	0.391
Female	Control (n=221)	TB (n=196)		
GG	137 (62.0%)	118 (60.2%)		
GA	63 (28.5%)	73 (37.2%)		
AA	21 (9.5%)	5 (2.6%)	0.25 (0.09-0.68)	0.0023

*Odd ratio (OR) and *p* values were calculated for the comparison of AA vs. GG+GA.

### Association between *NCF1* rs201802880 and clinical features of TB

The observed association of the NCF1 rs201802880 variant with TB susceptibility in women prompted an investigation into its relationship with clinical features of TB. We compared patients with different genotypes (GG+GA vs AA) regarding clinical presentation, treatment responses, and follow-up outcomes. As detailed in **Table 3**, the two patient subgroups were comparable in terms of age, PPD test positivity, erythrocyte sedimentation rate (ESR), treatment response, drug resistance, and most hematological parameters. A significant difference was noted in platelet counts, with patients carrying the AA genotype exhibiting higher platelet levels compared to those with GG or GA genotypes (295.6 ± 107.9 vs 241.5 ± 79.5, *p* = 0.004). Additionally, although not statistically significant, there was a trend towards a lower rate of extrapulmonary tuberculosis (0.00% vs 8.32%) and a reduced female-to-male ratio (5/18 vs 191/278) in patients with the AA genotype (**Table 3**). Gender-stratified analysis did not reveal any additional difference between the two patient subgroups (**Supplementary Table 2**).

**Table 3:** Association of NCF1 Arg90His variation with clinical and immunological characteristics in patients with TB.

	GG + GA (n=469)	AA (n=23)
Age, years (mean ± SD)	45.0 ± 18.7	43.5 ± 17.8
Sex (female/male)	191/278	5/18
Extrapulmonary tuberculosis	39 (8.32%)	0 (0.00%)
PPD above 10 mm	194/209 (92.8%)	13/14 (92.9%)
ESR (mm/h)	20 (7 - 44)	31 (13.5 - 55.5)
Treatment	469 (100%)	23 (100%)
Response to treatment	378 (80.5%)	18 (78.2%)
Drug resistance	97 (20.7%)	4 (17.4%)
Hematological parameters		
WBC (10^3^/μL)	6.31 ± 2.19	6.87 ± 2.50
NEU (10^3^/μL)	4.07 ± 1.94	4.69 ± 2.43
EOS (10^3^/μL)	0.18 ± 0.17	0.17 ± 0.13
BAS (10^3^/μL)	0.03 ± 0.02	0.03 ± 0.02
MON (10^3^/μL)	0.42 ± 0.17	0.46 ± 0.17
LYM (10^3^/μL)	1.59 ± 0.59	1.52 ± 0.57
RBC (10^6^/μL)	4.64 ± 0.55	4.83 ± 0.38
PLT (10^3^/μL)	241.5 ± 79.5	295.6 ± 107.9*

Quantitative data following a normal distribution are expressed as mean ± standard deviation (SD), whereas non-normally distributed quantitative data are reported as median (Q1–Q3). Categorical variables are presented as frequency (number of samples) and percentage. *p<0.05.

### Association between *NCF1* rs201802880 and neutrophil counts in healthy subjects

**Figure 1:**
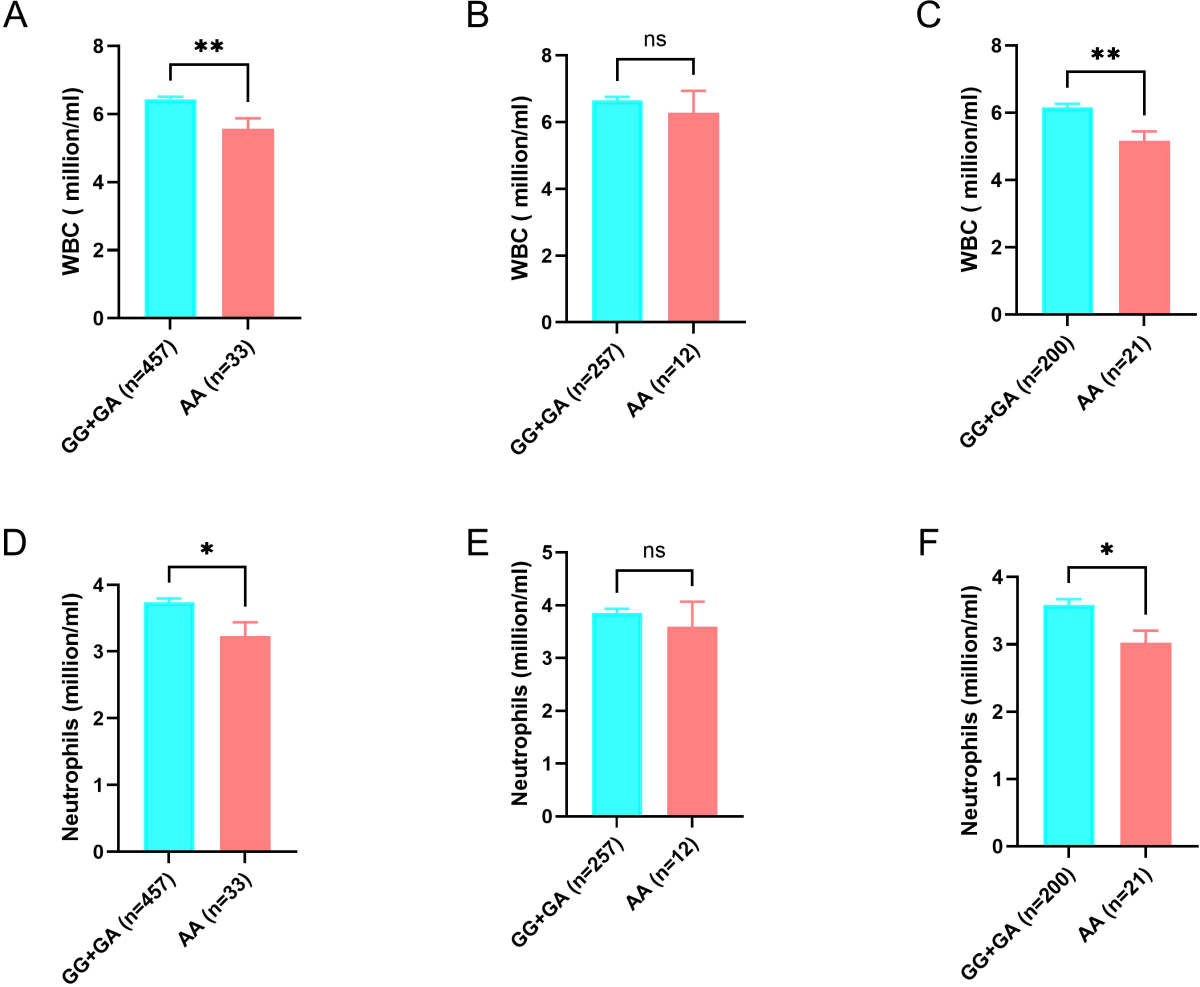
Association of NCF1 rs201802880 with circulating levels of white blood cells (WBC) and neutrophils in healthy control subjects. Comparison of levels of circulating WBC between AA and GG+GA genotypes in all subject **(A)**, men **(B)** and women **(C)**. Comparison of levels of circulating neutrophils between AA and GG+GA genotypes in all subject **(D)**, men **(E)** and women **(F)**. Data are presented as mean ± SEM. Statistical significance was determined using unpaired student’s t test. ns, not significant, *p<0.05 and **p<0.01.

Given the substantial alterations in hematological parameters during Mtb infection, we next examined whether the NCF1 His90 variant is associated with hematological parameters in healthy subjects. Compared to those with GG or GA genotypes, individuals with the AA genotype had significantly lower white blood cell counts (5.56 ± 1.78 vs 6.43 ± 1.59, *p* = 0.003). This difference was primarily due to lower neutrophil counts in AA genotype carriers compared to GG+GA carriers (3.23 ± 1.20 vs 3.74 ± 1.23, *p* = 0.02), with no significant differences observed in other leukocyte types (**Figure 1**, **Table 4**). Additionally, healthy individuals with the AA genotype exhibit lower platelet counts compared to those with the GG or GA genotypes (**Table 4**). Stratified analysis by gender revealed that the reduction in circulating white blood cells and neutrophils associated with the AA genotype was more pronounced in women compared to men (**Figure 1**, **Supplementary Table 3**).

**Table 4:** Association of NCF1 Arg90His variation with laboratory characteristics in healthy subjects.

	GG + GA (n=457)	AA (n=33)
Age, years (mean ± SD)	38.4 ± 15.8	42.8 ± 14.8
Sex (female/male)	200/257	21/12*
Hematological parameters		
WBC (10^3^/μL)	6.43 ± 1.59	5.56 ± 1.78**
NEU (10^3^/μL)	3.74 ± 1.23	3.23 ± 1.20*
EOS (10^3^/μL)	0.14 ± 0.12	0.12 ± 0.10
BAS (10^3^/μL)	0.03 ± 0.02	0.03 ± 0.02
MON (10^3^/μL)	0.36 ± 0.18	0.33 ± 0.12
LYM (10^3^/μL)	2.14 ± 0.65	2.00 ± 0.51
RBC (10^6^/μL)	4.79 ± 0.57	4.66 ± 0.73
PLT (10^3^/μL)	244.4 ± 63.2	222.6 ± 59.4*

Quantitative data following a normal distribution are expressed as mean ± standard deviation (SD), whereas non-normally distributed quantitative data are reported as median (Q1–Q3). Categorical variables are presented as frequency (number of samples) and percentage. * *p*<0.05, **p<0.01.

## Discussion

In this study, we investigated the association between the autoimmune disease-causal variant NCF1 rs201802880 A and tuberculosis, a persistent global infectious disease. Our findings suggest that the AA genotype of the NCF1 rs201802880 polymorphism is associated with a protective effect against active TB in women. Furthermore, the AA genotype correlates with reduced levels of white blood cells and neutrophils in healthy individuals. To date, three genome-wide association studies (GWAS) have been conducted to investigate TB susceptibility in Chinese populations, identifying more than ten genetic loci associated with the disease (19–21). However, the genetic region encompassing the *NCF1* gene on chromosome 7 has not been identified as a susceptibility locus for TB. This discrepancy may be attributed to the fact that all prior GWAS analyses were based solely on allele frequencies and did not incorporate stratified analyses by sex (19–21). Given that only the *NCF1* AA genotype, rather than the *NCF1* A allele, is associated with TB in women, it is unsurprising that this genetic association was not detected in previous studies.

The NCF1 rs201802880 A variant, while conferring protection against TB, is associated with an increased susceptibility to various autoimmune disorders, exemplifying evolutionary trade-offs (22). Throughout human history, our immune system has evolved under the selective pressure of infectious diseases such as TB, which posed significant threats to survival (23). Consequently, genetic variants that enhanced resistance to infections were positively selected. In modern contexts, where infectious diseases are less prevalent, these same variants may lead to overactive immune responses and contribute to autoimmune disorders (24).

Notably, the AA genotype of NCF1 rs201802880, which confers protection against TB in women, is linked to decreased circulating neutrophil levels in healthy individuals. This decrease is more pronounced in women than in men. Neutrophils, the most abundant leukocytes in the blood, play a crucial role in the early immune response to *Mtb* infection (25). In both human TB and animal models, lung disease manifestations are characterized by neutrophilic inflammation (25, 26), highlighting the critical role of neutrophils in TB pathogenesis.

Neutrophils are thought to play a dual role in the development of TB (27). On one hand, they are highly efficient pathogen-killing cells, employing both direct and indirect mechanisms to contribute significantly to the clearance of *Mtb* infection. On the other hand, neutrophils have been implicated in promoting *Mtb* growth and facilitating TB progression. For instance, studies have demonstrated that the risk of TB infection is inversely and independently associated with peripheral blood neutrophil count (28). Additionally, CXCL5 deficiency in murine TB models results in resistance to *Mtb* infection, attributed to impaired neutrophil recruitment from the bloodstream (29). Thus, it is plausible that the NCF1 rs201802880 AA genotype may confers protection against TB, at least in part, by reducing circulating neutrophil levels.

Both human studies and animal experiments have demonstrated that the NCF1 rs201802880 AA genotype results in reduced ROS production in neutrophils (9, 11, 12). While ROS are essential for neutrophil-mediated elimination of *Mtb* (30), pathogenic *Mtb* strains can exploit ROS to enhance their survival (31). For instance, it has been observed that virulent *Mtb* can persist within human neutrophils despite their rapid activation. This survival is associated with the necrotic death of infected neutrophils, a process entirely dependent on ROS production (32). Therefore, while a complete deficiency in neutrophil-derived ROS impairs the clearance of *Mtb*, a moderate reduction in neutrophil ROS production may paradoxically confer resistance to TB development.

Notably, the AA genotype is associated with TB specifically in women, but not in men. This sex-specific association suggests that the effect of the *NCF1* variant is more pronounced in females than in males. Experimental evidence from knock-in mice supports this hypothesis, as female mice carrying the *NCF1* AA genotype exhibit splenomegaly, increased IFN scores, the development of autoantibodies, and lupus-like kidney disease following pristane injection. In contrast, male mice with the same genotype show no evidence of autoimmune disease manifestation (11). The present study further supports this notion, as the decrease in circulating neutrophil levels associated with the AA genotype is more pronounced in women than in men.

Based on these findings, we propose a hypothetical mechanism for the protective effect of the NCF1 rs201802880 AA genotype against TB infection in women. The AA genotype may lead to lower circulating neutrophil levels, thereby impairing neutrophil recruitment to the lung and reducing the transition from *Mtb* infection to active TB and subsequent neutrophil-mediated tissue damage. Additionally, decreased ROS production in neutrophils may prevent Mtb-triggered ROS-dependent necrotic cell death and facilitate bacterial elimination. It is important to note that this is a simplified model, and other mechanisms involving various immune cells, such as macrophages and dendritic cells, and their dysregulation may also contribute to the observed protective effect against TB (9, 11, 12). For instance, the NCF1 variant may contribute to TB development by affecting the function of antigen-presenting cells. Evidence indicates that NADPH oxidase regulates the activity of cysteine cathepsins by modulating the lumenal redox potential, thereby influencing the production of the MHC II repertoire. This, in turn, impacts antigen presentation and CD4+ T cell-mediated immunity (33, 34). Therefore, it is plausible that the NCF1 AA genotype, which impairs ROS production, may alter the presentation of *Mtb* antigens to CD4^+^ T cells. Given the pivotal role of CD4^+^ T cells in controlling *Mtb* infection (35), it is reasonable to propose that the NCF1 variant confers resistance to TB in women by modulating antigen presentation.

This study has two main limitations. Firstly, the association between the NCF1 rs201802880 polymorphism was examined in a single case-control study, and the lack of replication compromises the robustness of the findings. Secondly, although the sample size is substantial, it may still be insufficient for stratified analyses. For example, examining associations between the NCF1 rs201802880 polymorphism and clinical features of TB in subgroups with relatively small sample sizes may reduce statistical power. Therefore, further validation in independent case-control studies with larger sample sizes is warranted.

In conclusion, this study is the first to demonstrate that the autoimmune disease-causal NCF1 variant is associated with a protective effect against TB infection. This finding exemplifies the evolutionary trade-offs where genetic variations that were positively selected for protection against infectious diseases may also increase the risk of autoimmune disorders.

## Data Availability

The original contributions presented in the study are included in the article/**Supplementary Material**. Further inquiries can be directed to the corresponding authors.
