# Supplementary material for: The autoimmune disease risk variant NCF1-His90 is associated with a reduced risk of tuberculosis in women
Source: Front Immunol. 2025 Jan 23;16:1514296. doi: 10.3389/fimmu.2025.1514296 (PMC11799249; doi:10.3389/fimmu.2025.1514296)
Supplement: Supplementary file 1 [file Table1.docx]

**Supplementary information**

**Table S1. Analysis of association between NCF1 Arg90His variation with TB with five genetic models**

|  | Model | Genotype | Controls (n=490) | TB (n=492) | OR (95% CI) | *P value* | AIC | BIC |
| --- | --- | --- | --- | --- | --- | --- | --- | --- |
| All subjects | Codominant | GG | 318 (64.9%) | 300 (61.0%) |  | 0.11 | 1362.1 | 1376.8 |
|  |  | GA | 139 (28.4%) | 169 (34.3%) | 1.29 (0.98-1.70) |  |  |  |
|  |  | AA | 33 (6.7%) | 23 (4.7%) | 0.68 (0.39-1.17) |  |  |  |
|  | Dominant | GG | 318 (64.9%) | 300 (61.0%) |  | 0.20 | 1363.7 | 1373.5 |
|  |  | GA+AA | 172(35.1%) | 192 (39.0%) | 1.18 (0.91-1.53) |  |  |  |
|  | Recessive | GG+GA | 457 (93.3%) | 469 (95.3%) |  | 0.16 | 1363.4 | 1373.2 |
|  |  | AA | 33 (6.7%) | 23 (4.7%) | 0.68 (0.39-1.17) |  |  |  |
|  | Overdominant | GG+AA | 351 (71.6%) | 323 (65.7%) |  | 0.04 | 1361.3 | 1371 |
|  |  | GA | 139 (28.4%) | 169 (34.3%) | 1.32 (1.01-1.73) |  |  |  |
|  | Log-additive | --- | --- | --- | 1.05 (0.85-1.30) | 0.63 | 1365.1 | 1374.9 |
| Male subjects | Codominant | GG | 181 (67.3%) | 182(61.5%) |  | 0.32 | 785.7 | 798.7 |
|  |  | GA | 76(28.3%) | 96 (32.4%) | 1.26 (0.87-1.81) |  |  |  |
|  |  | AA | 12 (4.4%) | 18 (6.1%) | 1.49 (0.70-3.19) |  |  |  |
|  | Dominant | GG | 181 (67.3%) | 182(61.5%) |  | 0.15 | 783.9 | 792.6 |
|  |  | GA+AA | 88(32.7%) | 114(38.5%) | 1.29 (0.91-1.82) |  |  |  |
|  | Recessive | GG+GA | 257 (95.6%) | 278(93.9%) |  | 0.39 | 785.2 | 793.9 |
|  |  | AA | 12 (4.4%) | 18 (6.1%) | 1.39 (0.66-2.94) |  |  |  |
|  | Overdominant | GG+AA | 193 (71.7%) | 200 (67.6%) |  | 0.28 | 784.8 | 793.5 |
|  |  | GA | 76(28.3%) | 96 (32.4%) | 1.22 (0.85-1.75) |  |  |  |
|  | Log-additive | --- | --- | --- | 1.24 (0.93-1.64) | 0.13 | 783.7 | 792.4 |
| Female subjects | Codominant | GG | 137 (62.0%) | 118(60.2%) |  | 0.0036 | 571.3 | 583.4 |
|  |  | GA | 63(28.5%) | 73(37.2%) | 1.35 (0.89-2.04) |  |  |  |
|  |  | AA | 21 (9.5%) | 5 (2.6%) | 0.25 (0.09-0.68) |  |  |  |
|  | Dominant | GG | 137 (62.0%) | 118(60.2%) |  | 0.709 | 580.4 | 588.5 |
|  |  | GA+AA | 84(38.0%) | 78(39.8%) | 1.08 (0.73-1.60) |  |  |  |
|  | Recessive | GG+GA | 200 (90.5%) | 191 (97.4%) |  | 0.0023 | 571.3 | 579.4 |
|  |  | AA | 21 (9.5%) | 5 (2.6%) | 0.25 (0.09-0.68) |  |  |  |
|  | Overdominant | GG+AA | 158 (71.5%) | 123 (62.8%) |  | 0.058 | 577 | 585 |
|  |  | GA | 63(28.5%) | 73(37.2%) | 1.49 (0.99-2.25) |  |  |  |
|  | Log-additive | - | - |  | 0.87 (0.63-1.19) | 0.39 | 579.8 | 587.9 |

Five inheritance models—co-dominant, dominant, recessive, over-dominant, and additive—were applied for the genetic association analysis using the SNPSTATS program (<https://www.snpstats.net/>). The optimal inheritance model was determined based on the Akaike Information Criterion (AIC) and Bayesian Information Criterion (BIC), with the model yielding the lowest AIC and BIC values considered the best fit.

**Table S2. Gender-stratified analysis of association of NCF1 Arg90His variation with clinical and immunological characteristics in TB patients**

|  | Male | | Female | |
| --- | --- | --- | --- | --- |
|  | GG + GA (n=278) | AA (n=18) | GG + GA (n=191) | AA (n=5) |
| Age, years | 45.9 ± 18.3 | 44.8 ± 18.2 | 44.0 ± 19.1 | 39.0 ± 17.5 |
| Extrapulmonary tuberculosis | 18 (8.32%) | 0 (0.00%) | 21 (10.9%) | 0 (0.00%) |
| PPD above 10 mm | 110/119 (92.4%) | 8/9 (88.8%) | 84/90 (93.3%) | 5/5 (100%) |
| ESR, mm/h | 20 (6 - 40) | 31 (13.5 - 55.5) | 21 (8 - 48.3) | 31 (11 - 90) |
| Treatment | 278 (100%) | 18 (100%) | 278 (100%) | 18 (100%) |
| Response to treatment | 207 (74.5%) | 14 (77.7%) | 171 (89.5%) | 4 (80%) |
| Drug resistance | 76 (27.3%) | 4 (22.2%) | 21 (11.0%) | 0 (0%) |
| Haematological parameters |  |  |  |  |
| WBC (10^3^/μL) | 6.70 ± 2.19 | 6.76 ± 2.17 | 5.76 ± 2.09 | 7.19 ± 3.63 |
| NEU (10^3^/μL) | 4.39 ± 1.89 | 4.45 ± 2.01 | 3.63 ± 1.94 | 5.39 ± 3.63 |
| EOS (10^3^/μL) | 0.20 ± 0.18 | 0.19± 0.14 | 0.15 ± 0.15 | 0.12 ± 0.08 |
| BAS (10^3^/μL) | 0.04 ± 0.02 | 0.03 ± 0.01 | 0.03 ± 0.02 | 0.04 ± 0.02 |
| MON (10^3^/μL) | 0.46 ± 0.18 | 0.49 ± 0.17 | 0.35 ± 0.13 | 0.39 ± 0.16 |
| LYM (10^3^/μL) | 1.59 ± 0.62 | 1.60 ± 0.61 | 1.59 ± 0.56 | 1.26 ± 0.33 |
| RBC (10^6^/μL) | 4.78 ± 0.60 | 4.92 ± 0.32 | 4.45 ± 0.42 | 4.55 ± 0.45 |
| PLT (10^3^/μL) | 235.9 ± 81.4 | 301.7 ± 109.4** | 249.3 ± 76.3 | 238.8 ± 113.2 |

Quantitative data following a normal distribution are expressed as mean ± standard deviation (SD), whereas non-normally distributed quantitative data are reported as median (Q1–Q3). Categorical variables are presented as frequency (number of samples) and percentage. Comparisons were conducted between the AA genotype and the combined GG+GA genotypes. ***p*<0.01.

**Table S3. Gender-stratified analysis of aassociation of NCF1 Arg90His variation with laboratory characteristics in healthy subjects**

|  | Male | | Female | |
| --- | --- | --- | --- | --- |
|  | GG + GA (n=257) | AA (n=12) | GG + GA (n=200) | AA (n=21) |
| Age, years (mean±SD) | 37.2 ± 15.2 | 35.0 ± 12.0 | 40.0 ± 16.5 | 47.4 ± 14.7* |
| Haematological parameters |  |  |  |  |
| WBC (10^3^/μL) | 6.65 ± 1.65 | 6.28 ± 2.28 | 6.16 ± 1.52 | 5.16 ± 1.31** |
| NEU (10^3^/μL) | 3.86 ± 1.25 | 3.60 ± 1.63 | 3.58 ± 1.19 | 3.02 ± 0.83* |
| EOS (10^3^/μL) | 0.16 ± 0.13 | 0.13 ± 0.09 | 0.12 ± 0.10 | 0.11 ± 0.11 |
| BAS (10^3^/μL) | 0.04 ± 0.02 | 0.04 ± 0.02 | 0.03 ± 0.03 | 0.03 ± 0.02 |
| MON (10^3^/μL) | 0.39 ± 0.19 | 0.36 ± 0.13 | 0.33 ± 0.15 | 0.30 ± 0.11 |
| LYM (10^3^/μL) | 2.14 ± 0.65 | 2.00 ± 0.51 | 2.12 ± 0.64 | 1.83 ± 0.52 |
| RBC (10^6^/μL) | 5.05 ± 0.55 | 5.22 ± 0.41 | 4.48 ± 0.40 | 4.33 ± 0.68 |
| PLT (10^3^/μL) | 239.8 ± 56.2 | 212.2 ± 62.2 | 252.6 ± 71.5 | 228.7 ± 58.4 |

Quantitative data following a normal distribution are expressed as mean ± standard deviation (SD), whereas non-normally distributed quantitative data are reported as median (Q1–Q3). Categorical variables are presented as frequency (number of samples) and percentage. Comparisons were conducted between the AA genotype and the combined GG+GA genotypes. ******p*<0.05, ***p*<0.01.
